# Exposure to Pre- and Perinatal Risk Factors Partially Explains Mean Differences in Self-Regulation between Races

**DOI:** 10.1371/journal.pone.0141954

**Published:** 2016-02-16

**Authors:** J. C. Barnes, Brian B. Boutwell, J. Mitchell Miller, Rashaan A. DeShay, Kevin M. Beaver, Norman White

**Affiliations:** 1 School of Criminal Justice, The University of Cincinnati, Cincinnati, OH, United States of America; 2 Criminology & Criminal Justice, School of Social Work, College for Public Health & Social Justice, Saint Louis University, St. Louis, MO, United States of America; 3 Department of Epidemiology (Secondary Appointment), College for Public Health & Social Justice, Saint Louis University, St. Louis, MO, United States of America; 4 Department of Criminology and Criminal Justice, University of North Florida, Jacksonville, FL, United States of America; 5 Department of Criminal Justice, California State University, Stanislaus, Turlock, CA, United States of America; 6 College of Criminology and Criminal Justice, Florida State University, Tallahassee, FL, United States of America; Shanghai 1st Maternity and Infant hospital of Tongji University, CHINA

## Abstract

**Objectives:**

To examine whether differential exposure to pre- and perinatal risk factors explained differences in levels of self-regulation between children of different races (White, Black, Hispanic, Asian, and Other).

**Methods:**

Multiple regression models based on data from the Early Childhood Longitudinal Study, Birth Cohort (*n ≈* 9,850) were used to analyze the impact of pre- and perinatal risk factors on the development of self-regulation at age 2 years.

**Results:**

Racial differences in levels of self-regulation were observed. Racial differences were also observed for 9 of the 12 pre-/perinatal risk factors. Multiple regression analyses revealed that a portion of the racial differences in self-regulation was explained by differential exposure to several of the pre-/perinatal risk factors. Specifically, maternal age at childbirth, gestational timing, and the family’s socioeconomic status were significantly related to the child’s level of self-regulation. These factors accounted for a statistically significant portion of the racial differences observed in self-regulation.

**Conclusions:**

The findings indicate racial differences in self-regulation may be, at least partially, explained by racial differences in exposure to pre- and perinatal risk factors.

## Introduction

Self-regulation—which can been defined as the regulation of the self by the self [1]—is a human phenotype that has a pronounced influence on a wide range of outcomes across the entire life course. The inability to regulate one’s attention in early childhood is a harbinger of maladaptive and problematic outcomes later in life [2–6]. Children who have problems with self-regulation are, for example, more likely to develop and manifest behavioral problems, to display signs of conduct disorder, and to have difficulties in forging social relationships [3, 7–8]. Children and adolescents who lack self-control—a phenotype that is closely related to self-regulation—are at risk for engaging in delinquent acts, for using and abusing drugs and alcohol, and for performing poorly in school [9–10]. Moreover, problems with self-control also affect economic success, overall health, and the probability of coming into contact with the criminal justice system in adulthood [4, 11]. Taken together, the available literature suggests self-regulation is an important trait that has consistent and wide-sweeping effects on a number of human complex traits.

Research has revealed that individual differences in self-regulation emerge within the first few years of life [12–15] and remain relatively stable throughout adolescence and adulthood [16]. As a result, there has been a significant amount of research devoted to uncovering the etiological origins of self-regulation. This rapidly expanding literature has revealed that a range of factors, including genetic/biological influences [17–19], cultural/social forces [18, 20], and school-based elements [21] influence the development of self-regulation during the first two decades of the life course. Although a number of disciplinary perspectives have been employed to explain the development of self-regulation, one perspective in particular that has generated some empirical support is the public health approach. This approach has centered on examining an array of factors, especially pre- and perinatal risk factors, and how they affect the development of self-regulation and related phenotypes [22–25].

Importantly, scholars have noted that mean levels of self-regulation differ across racial categories with Black respondents tending to score higher on measures of impulsivity compared to Asians and Whites [6, 26–27]. Additionally, there is evidence of differences in a range of temperament scores cutting across samples of Asian and American respondents [28–29]. In studies examining related traits—such as general intelligence (which is associated with long term planning, problem solving ability, increased prosocial behavior, and increased self-regulation)—similar race-graded patterns have emerged such that Blacks and Hispanics tend to evince lower scores than Whites and Asians [26–27, 30–31].

Given these findings, an intriguing question that has yet to be fully addressed concerns the degree to which exposure to pre- and perinatal risk factors explains racial disparities in measures of self-regulation (see, generally [32–34]). While few studies have addressed this question, the literature base offers two general explanations of any observed racial differences in self-regulation [35–36]. First, racial differences in mean levels of self-regulation may be due, in part, to differential exposure to risk factors (i.e., an exposure-level hypothesis). If one group is more likely to experience trait-relevant risk factors than another group, then the former should exhibit lower levels of self-regulation on average. We refer to this explanation as Hypothesis 1.

A second explanation also highlights the importance of risk factors, but suggests that groups differ in their susceptibility to risk. Thus, racial groups may exhibit mean differences in self-regulation because one group is differentially more or less vulnerable to the effects of the risk factors. This moderating explanation posits a statistical interaction between risk factors and race in the prediction of self-regulation. We refer to this explanation as Hypothesis 2. It is also important to note that Hypothesis 2—the moderation explanation—is consistent with the argument that certain experiences (e.g., education) qualitatively differ across racial lines. If this were the case, we might expect some factors to matter more for one group compared to another [37]. (An anonymous reviewer deserves credit for raising the possibility that Hypothesis 2 may be supported if *interpretations* of the experiences differ across racial groups.)

While relatively little research has considered Hypothesis 2, there is some evidence to support Hypothesis 1; that is, that racial groups differ in their level of exposure to risk factors [32–33, 36]. Indeed, Lynch [38] reported a wide range of racial disparities in early child health and development using data from the Early Childhood Longitudinal Study, Birth Cohort (the same data that will be analyzed here). Moreover, national statistics show that of the four million births in 2001, approximately 83 percent received prenatal care during the first trimester, meaning that roughly 17 percent did not receive such medical attention. Breaking down this statistic by racial categories revealed that approximately 89 percent of White mothers received prenatal care during the first trimester while approximately 75 percent of Black mothers, 69 percent of American Indian mothers, 84 percent of Asian or Pacific Islander mothers, and 76 percent of Hispanic mothers received such care [39]. (The analysis presented below utilizes data drawn from the year 2001, so we present national statistics from that same year.) This finding leaves open the possibility that minority mothers are more likely to experience pregnancy and/or birth complications than White mothers due to their lesser access to, or utilization of, prenatal care. Racial differences were also observed for other indicators such as maternal age at childbirth, a purported risk factor for the child’s development [40], and length of the gestation period (e.g., Black children tended to be born earlier than other children [39]; see also [36]).

## Methods

### Sample

Respondents were drawn from the Early Childhood Longitudinal Study, Birth Cohort (ECLS-B). A description of the sampling design is available elsewhere [41]. Briefly, the ECLS-B is a large, nationally representative sample of almost all children born in the United States in 2001. More than 10,000 children were entered into the study at wave 1 and a large majority was retained over the subsequent waves of data collection (ECLS-B privacy policy requires that all sample sizes/case counts be rounded to the nearest 50, so all sample sizes reported in this study have been rounded to avoid deductive disclosure). To date, five waves of data have been collected and are available for analysis. Wave 1 was collected when the children were approximately 9 months of age, wave 2 was collected when the children were approximately 2 years old, wave 3 was collected when the children were approximately 4 years old, wave 4 was collected when the children had reached 5 years of age, and wave 5 was collected when the children entered kindergarten.

### Dependent Variable: Self-Regulation

Several items adopted from the Infant/Toddler Symptom Checklist (ITSC [42]) were utilized as a measure of self-regulation. During wave 2 interviews, parents in the study were asked seven questions related to the frequency with which their child was irritable or fussy, went from a whimper to a cry, was unable to wait without crying, was easily distracted, needed help falling asleep, tuned out from activity, and had problems focusing on a given task. Exploratory and confirmatory factor analysis suggested the seven items could be combined into a single scale because all of the items loaded onto a single latent factor. Thus, responses were summed to create a self-regulation scale that ranged from 1 (indicating low self-regulation) to 22 (indicating high self-regulation) (α = 0.67). The unweighted mean (survey weights will be used for the multiple regression analysis presented below) for the scale prior to any data imputation (multiple imputation will be used in the analysis) was 13.01 with a standard deviation of 4.29. A correlation matrix between the self-regulation scale and all of the risk factors presented below is provided in S1 Table.

### Primary Predictor Variable: Race

Information used to identify the child’s race was collected from the parent interview at wave 1. Parents were given 14 racial categories and were asked to select the category (ies) to which their child belonged. Information about the child’s Hispanic or Latino ethnicity was also collected. Any parent who identified their child as coming from a Hispanic or Latino heritage was automatically coded as *Hispanic* (= 1). From these pieces of information, ECLS-B staff researchers constructed a composite indicator that originally contained eight racial categories: *White (non-Hispanic)*, *Black or African American (non-Hispanic)*, *Hispanic (and other racial category specified)*, *Hispanic (no other racial category specified)*, *Asian (non-Hispanic)*, *Native Hawaiian or other Pacific Island*, *American Indian or Alaska Native (non-Hispanic)*, *and more than one race (non-Hispanic)*. Five racial categories were created for the purposes of this analysis: *White (non-Hispanic)* (*n ≈* 4,450), *Black or African American (non-Hispanic)* (*n ≈* 1,700), *Hispanic* (which includes those who selected a racial category and those who did not select a racial category) (*n ≈* 2,200), *Asian (non-Hispanic)* (*n ≈* 1,200), and *Other* (which includes *Native Hawaiian or other Pacific Island*, *American Indian or Alaska Native*, and those who selected more than one racial category) (*n ≈* 1,100).

### Risk Factors

#### Maternal Age at Childbirth

Maternal age at childbirth may be a salient risk factor for the development of self-regulation in early childhood [43]. Thus, a single item assessing maternal age (drawn from the child’s birth certificate) was included in the analysis. The unweighted mean for the entire sample prior to imputation was 27.49 with a standard deviation of 6.36.

#### Maternal Education at Childbirth

Maternal education at childbirth may be an important factor for self-regulation development. In order to account for these effects, the mother’s level of education at the time of childbirth (coded as years in school) was included. The unweighted mean for the entire sample prior to imputation was 13.04 with a standard deviation of 2.81.

#### Prenatal Care

Mothers who receive prenatal care have children who are, on average, less likely to suffer from adverse pregnancy outcomes [35, 44–45] and are less likely to have children who are involved in serious antisocial behavior [46]. Thus, prenatal care may affect the child’s development of self-regulation. Information from the child’s birth certificate indicated the number of prenatal visits that were made by the mother while pregnant. Values ranged from 0 (none) to 49 (indicating 49 or more visits). The unweighted mean for the entire sample prior to imputation was 11.38 with a standard deviation of 4.40. The large majority of cases (98%) ranged between 0 and 20 visits. As a sensitivity test, the *prenatal care* variable was recoded so that all values over 20 were coded as 20. The substantive findings gleaned from the analysis did not appear to be sensitive to this alternative coding scheme.

#### Gestation Week

Information regarding gestation period was gleaned from the child’s birth certificate. Values were coded as a discrete variable reflecting the number of weeks of gestation. This variable ranged between 17 and 47 with an unweighted mean for the entire sample prior to imputation of 37.38 and a standard deviation of 3.93.

#### Birth Weight

Children born of low birth weight are at increased risk of developing ADHD [47], which is to say that these children have a higher risk of developing problems with self-regulation. Birth weight was coded as a continuous variable measured in grams and ranged from 227 to 5,443. This information was gleaned from the child’s birth certificate. Birth weight was divided by 100 to make regression coefficients easier to interpret. The unweighted mean for the entire sample prior to imputation was 29.31 with a standard deviation of 8.81.

#### Apgar Score

Five-minute Apgar scores are a global measure used to assess the overall health, wellbeing, and development of infants shortly after birth. Apgar scores may be an indicator of the child’s development of self-regulation in early childhood. To account for this potential effect, the child’s Apgar score, which is denoted on the birth certificate, was included. Apgar scores ranged from a minimum of 0 to a maximum of 10. The unweighted mean for the entire sample prior to imputation was 8.76 with a standard deviation of 0.90.

#### Plural Births

Being part of a plural birth (e.g., twins or triplets) is a risk factor for a number of birth complications [48]. To account for this effect, a count variable coded as the number of children born to the mother at the time of the child’s birth was included in the analysis (values ranged from 1 = singleton to 4 = quadruplets). This information was also taken from the child’s birth certificate. The unweighted mean for the entire sample prior to imputation was 1.17 with a standard deviation of 0.40.

#### Vaginal Delivery

Non-vaginal delivery may indicate a birth complication and, therefore, may act as a proxy for the existence of some underlying developmental problem (s). A dichotomous variable indicating whether the child was born vaginally (0 = no, 1 = yes) was included in the analysis. Information regarding the delivery method was gleaned from the child’s birth certificate. The unweighted proportion of births born vaginally for the entire sample prior to imputation was 0.66.

#### Eclampsia

Eclampsia is a condition that affects some mothers during pregnancy and is characterized by the presence of seizures. Mothers who suffer this condition may have children with increased risks for developmental delays and perhaps even delinquent behavior [25]. Information from the child’s birth certificate indicated whether the mother had experienced eclampsia (0 = no, 1 = yes). The unweighted proportion of cases experiencing eclampsia in the entire sample prior to imputation was 0.01.

#### Meconium

Meconium-stained amniotic fluid may portend later developmental complications for the child [49]. A dichotomous variable that indicated whether meconium was moderate or heavy during birth (0 = no, 1 = yes) was available on the child’s birth certificate. The unweighted proportion of cases experiencing moderate or heavy meconimum prior to imputation was 0.04.

#### Maternal Tobacco Use

Prenatal exposure to nicotine has been linked with myriad negative outcomes including health problems, developmental delays, and even criminality [29, 50]. A dichotomous variable indicated whether the mother used tobacco during her pregnancy (0 = no, 1 = yes). This information was available on the child’s birth certificate. The unweighted proportion of cases reporting tobacco use prior to imputation was 0.13.

#### Socioeconomic Status (SES)

The family’s socioeconomic status (SES) was assessed at wave 1 by the ECLS-B staff and was included with the data file as a normally distributed standardized scale. The scale was created by standardizing and combining information about the father/male guardian’s (if present) education, the father/male guardian’s occupation, the mother/female guardian’s (if present) education, the mother/female guardian’s occupation, and the household income. Occupations were coded according to the *Standard Occupational Classification Manual* [51] and prestige scores were created by referring to the average prestige score garnered from the 1989 General Social Survey and the average prestige scores from the 2000 Census occupational categories (see [52] for more detail about the ECLS-B coding strategies). The SES variable was coded so that higher values indicated higher SES and complete data were available for all cases in the sample (i.e., there were no missing data for the SES variable). The unweighted mean was -0.05 with a standard deviation of 0.86.

### Control Variables

Controls for the child’s age at wave 2 (coded in months; unweighted mean prior to imputation was 24.49 with a standard deviation of 1.31 and a range between 16.8 and 38.2) and the child’s gender (0 = female, 1 = male; 51% of the sample was male) were included in the multiple regression analyses.

## Analysis Plan

The analysis proceeded in three general steps. First, we analyzed the mean (or proportion/prevalence) score for each of the analytic variables across all five racial categories. For example, we began by estimating the mean self-regulation score for White children, Black children, Hispanic children, Asian children, and children classified in the Other racial category. The same process was carried out for the risk factors described above. Proportions are reported whenever a risk factor was coded as a dichotomous variable (i.e., *Vaginal delivery*, *Eclampsia*, *Meconium*, and *Maternal tobacco use*). In an effort to limit the probability of a false-positive result (see generally, [53]), all *p*-values were adjusted for the multiple comparisons made across racial categories according to the Bonferroni correction method.

The second step to the analysis was to estimate the impact of race on self-regulation *after* accounting for the influences of all risk factors. This step was carried out with a multiple regression model using the identity link function because, although the variable is coded as discrete counts, the distribution of scores for the self-regulation scale was approximately normal. Results from this step will inform Hypothesis 1, the exposure-level hypothesis. The hypothesis will be supported if racial differences in self-regulation are diminished after controls for the pre-/perinatal risk factors are entered into the equation. We analyzed five racial categories, so it was necessary to compare coefficients across the various racial groups. As before, Bonferroni corrections were carried out to reduce the probability of a Type I error.

The third step to the analysis tested Hypothesis 2, the susceptibility hypothesis. Recall that this hypothesis suggests there will be interactions between race and the risk factors in the prediction of self-regulation scores. To assess this possibility, we estimated the effect of the various Risk Factor X Race interactions with the multiple regression model.

Two final points must be made before moving to the results. First, all estimates—with the exception of the correlations presented in the supporting information (S1 Table) and the summary statistics provided in the text above—were corrected for the survey weights provided by the ECLS-B research staff. Additionally, standard errors were adjusted using the primary sampling unit and stratification information provided by the ECLS-B. For this reason, all estimates presented below are considered population parameter estimates that can be generalized to U.S. children born in 2001. Second, missing data were imputed for the multiple regression analyses using the full-information maximum likelihood (FIML) estimation routine available in *Stata 14*. FIML simultaneously estimates the parameters and the missing values for all variables included in the multiple regression equation, making it a convenient strategy for handling missing data [54]. This procedure rests on the assumption that data are missing at random (MAR), which is the same assumption made when using the other most common strategy for handling missing data, listwise deletion. Thus, under the assumption of MAR, the estimates will not be biased when data are imputed with ML [54–55]. Roughly 80 percent of all cases were either not missing any data or were only missing information on one variable. All but two of the analytic variables had missing data rates that were below 5%. The two exceptions were *Apgar score* (18.7% of cases missing) and *Maternal tobacco use* (14.9% of cases missing).

## Findings

Table 1 presents population parameter estimates for the mean scores (for the quantitative variables) and proportions (for the categorical variables) for each of the variables used in the analysis. The 95 percent confidence interval for each parameter estimate is provided in parentheses below the parameter estimate. Notice that six estimates are given for each variable: one for the full sample, one for White children, one for Black children, one for Hispanic children, one for Asian children, and one for children classified in the Other category. Looking first at the self-regulation scale, White children had the highest average scores and Black children had the lowest average scores. Hispanic, Asian, and Other children had average scores that fell in between the mean scores for White children and Black children. Note that the global *F*-test indicated at least one of the racial groups had a statistically different mean score when compared to the other group (s) (*F* = 12.48, *p*<0.01). Post-hoc *t*-tests (Bonferroni corrected) were estimated and the results are presented in the last column on the right. Two general patterns are evident. First, White children had statistically higher mean scores than Black children, Hispanic children, and Asian children. The means for White children and Other children did not statistically differ on the self-regulation scale. Second, Black children had lower mean scores on the self-regulation scale than White children, Hispanic children, and Other children. The means for Black children and Asian children did not differ (statistically) on the self-regulation scale. These findings reveal that self-regulation varies across racial categories, providing justification for testing Hypothesis 1 and Hypothesis 2.

**Table 1:** Mean Scores for ECLS-B Study Variables.

	All Children	White Children	Black Children	Hispanic Children	Asian Children	Other Children	*F*-test	Post-hoc *t*-test (w/Bonferroni Correction)
Self-regulation	13.263 (13.075–13.451)	13.635 (13.386–13.885)	12.301 (11.982–12.620)	13.072 (12.761–13.383)	12.788 (12.363–13.213)	13.110 (12.698–13.522)	12.48**	W>B**; W>H*; W>A**; B<H**; B<O*
Maternal Age at Childbirth	27.306 (27.026–27.586)	28.386 (27.971–28.802)	25.174 (24.745–25.602)	26.050 (25.699–26.401)	29.769 (29.317–30.221)	26.645 (26.016–27.274)	77.28**	W>B**; W>H**; W<A**; W>O**; B<H*; B<A**; B<O**; H<A**; A>O**
Maternal Eduation at Childbirth	12.884 (12.738–13.030)	13.798 (13.632–13.964)	12.398 (12.236–12.561)	11.006 (10.744–11.267)	14.161 (13.884–14.438)	12.997 (12.720–13.274)	148.52**	W>B**; W>H**; W>O**; B>H**; B<A**; B<O**; H<A**; H<O**; A>O**
Prenatal Care Visits	11.531 (11.353–11.708)	11.986 (11.786–12.187)	10.796 (10.385–11.206)	11.013 (10.715–11.311)	11.554 (11.233–11.874)	11.079 (10.669–11.488)	15.76**	W>B**; W>H**; W>O**; B<A*;
Gestation Week	38.754 (38.691–38.817)	38.849 (38.773–38.926)	38.360 (38.190–38.530)	38.801 (38.671–38.932)	38.827 (38.698–38.955)	38.531 (38.326–38.735)	8.91**	W>B**; W>O*; B<H**; B<A**;
Birth weight/100	33.160 (32.994–33.326)	33.772 (33.553–33.992)	31.246 (30.957–31.536)	33.107 (32.817–33.397)	31.712 (31.383–32.042)	32.799 (32.171–33.426)	76.91**	W>B**; W>H**; W>A**; W>O*; B<H**; B<O**; H>A**; A<O*
Apgar Score	8.933 (8.902–8.965)	8.935 (8.897–8.973)	8.881 (8.799–8.964)	8.982 (8.950–9.014)	8.932 (8.884–8.981)	8.898 (8.846–8.951)	2.65*	-
Plural Births	1.034 (1.032–1.036)	1.040 (1.037–1.043)	1.032 (1.027–1.038)	1.025 (1.021–1.028)	1.023 (1.016–1.029)	1.029 (1.021–1.036)	11.24**	W>H**; W>A**
Vaginal Delivery^a^	0.739 (0.725–0.753)	0.736 (0.719–0.753)	0.728 (0.693–0.760)	0.747 (0.720–0.772)	0.762 (0.727–0.794)	0.749 (0.699–0.793)	0.52	-
Eclampsia^a^	0.003 (0.002–0.004)	0.002 (0.001–0.005)	0.005 (0.002–0.011)	0.002 (0.001–0.007)	0.0003 (0.0001–0.003)	0.002 (0.001–0.010)	1.12	-
Meconium^a^	0.048 (0.042–0.056)	0.042 (0.033–0.052)	0.073 (0.057–0.094)	0.049 (0.038–0.063)	0.043 (0.030–0.062)	0.052 (0.034–0.078)	4.31**	W<B*
Maternal Tobacco Use^a^	0.127 (0.114–0.142)	0.159 (0.141–0.178)	0.087 (0.068–0.110)	0.059 (0.045–0.079)	0.010 (0.002–0.046)	0.213 (0.171–0.261)	30.72**	W>B**; W>H**; W>A**; B>A**; B<O**; H>A**; H<O**; A<O**
Socioeconomic Status W1	-0.079 (-0.126- -0.033)	0.202 (0.144–0.259)	-0.487 (-0.547- -0.426)	-0.499 (-0.551- -0.447)	0.487 (0.384–0.590)	-0.156 (-0.231- -0.080)	245.56**	W>B**; W>H**; W<A**; W>O**; B<A**; B<O**; H<A**; H<O**; A>O**

^a^ Proportions reported; All estimates corrected for the survey design features of the ECLS-B study; 95% confidence interval in parentheses;

*p <.05;

**p <.01 (two-tailed).

Turning next to the risk factors under examination, the last column on the right side of Table 1 reveals that 9 of the 12 risk factors evinced variability across the five racial groups. For instance, Black children were, on average, born to younger mothers than children from all other racial groups. Black mothers also had the lowest mean number of prenatal care visits compared to mothers of White children and Asian children. (The difference between Asian children and Hispanic children emerged as marginally significant (*p* = 0.044) when the *prenatal care* variable was capped at 20 visits [see above].) White children and Hispanic children had the highest mean birth weights of all five racial categories. White children were, however, more likely to be born to a mother who reported tobacco use compared to Black children, Hispanic children, and Asian children. Finally, socioeconomic status (SES) varied across the five groups. Only the difference between Black children and Hispanic children failed to reach statistical significance. When taken together, the findings presented in Table 1 reveal that many of the five racial categories differ in terms of their level of exposure to the various risk factors. It is noteworthy, however, that the five racial groups did *not* statistically differ in their Apgar scores (once the Bonferroni correction was made), in their prevalence of vaginal delivery, or in the proportion of births where the mother experienced eclampsia.

Given the sweeping racial differences that were noted in Table 1, the next step was to estimate whether the racial differences in mean self-regulation scores were attributable (at least partially) to the observed racial differences in risk factor exposure. Presented in Table 2 are the results from four regression equations. The first model, the baseline model, provides parameter estimates for the effect of race on self-regulation after controlling for the child’s age at wave 2 (in months, mean-centered) and the child’s sex. Note that White children served as the omitted category because they were estimated to have the highest levels of self-regulation on average (see Table 1). Thus, all of the coefficients for the racial categories presented in the baseline model of Table 2 are interpreted as mean differences in self-regulation for the racial category of focus compared to White children. Also, it is important to recall that missing data were imputed for the multiple regression models. Variables that are not explicitly modeled as part of the structural equation (i.e., the risk factors are not included in the baseline model) were used as auxiliary variables for the imputation procedure [54]. This will allow for the comparison of coefficients across the different models by ensuring that imputation was carried out in a consistent manner.

**Table 2:** OLS Regression of Self-regulation on Control Variables and Risk Factors (*n* ≈ 9,850).

	Model 1: Baseline Model	Model 2: Select Risk Factor Model	Model 3: Full Risk Factor Model	Model 4: Trimmed Risk Factor Model
	*b (*se)	*b (*se)	*b (*se)	*b (*se)
*Primary Predictor Variables*				
Black = 1	-1.338** (0.186)	-1.006** (0.198)	-0.847** (0.224)	-0.854** (0.222)
Hispanic = 1	-0.581**(0.178)	-0.219(0.187)	-0.140(0.192)	-0.168(0.195)
Asian = 1	-0.855**(0.236)	-0.999**(0.229)	-1.069**(0.226)	-1.108**(0.225)
Other = 1	-0.537*(0.227)	-0.323(0.221)	-0.257(0.225)	-0.252(0.224)
*Control Variables*				
Age W2	0.101(0.057)	0.125*(0.059)	0.127*(0.059)	0.125*(0.059)
Male = 1	-0.420**(0.124)	-0.416**(0.125)	-0.428**(0.126)	-0.418**(0.124)
*Risk Factors*				
Maternal Age at Childbirth	--	0.054**(0.014)	0.043**(0.013)	0.045**(0.013)
Maternal Education at Childbirth	--	0.100**(0.025)	0.028(0.033)	--
Prenatal Care Visits	--	-0.011(0.016)	-0.013(0.016)	--
Gestation Week	--	0.040(0.030)	0.041(0.030)	0.057*(0.023)
Birth weight/100	--	0.015(0.011)	0.014(0.011)	--
Apgar	--	0.067(0.092)	0.062(0.091)	--
Plural Births	--	0.117(0.181)	0.092(0.180)	--
Vaginal Delivery = 1	--	0.233(0.148)	0.212(0.146)	0.214(0.143)
Eclampsia = 1	--	-0.343(0.780)	-0.396(0.763)	--
Meconium = 1	--	0.100(0.259)	0.124(0.260)	--
Maternal Tobacco Use = 1	--	-0.405(0.223)	-0.294(0.224)	-0.322(0.224)
Socioeconomic Status W1	--	--	0.424**(0.135)	0.487**(0.102)

Standard errors in parentheses; All estimates corrected for the survey design features of the ECLS-B study;

*p <.05;

**p <.01 (two-tailed).

Looking at the results from the baseline model reveals that three of the mean differences between White children and the other racial categories remain after controlling for age and sex. Indeed, the coefficients for *Black* (*p*<0.0001), *Hispanic* (*p* = 0.0092), and for *Asian* (*p* = 0.0029) were negative and statistically significant after carrying out a Bonferroni correction. Although the *Other* coefficient is labeled as statistically significant in Table 2, consistent with the results from Table 1, the mean difference between White children and Other children was not statistically significant after a Bonferroni correction was carried out (*p* = .121). Recall, however, that the results in Table 1 indicated that Black children had lower mean scores on the self-regulation scale when compared to Hispanic children and Other children. These comparisons are not readily available in the results presented in Table 2, so a series of post-hoc tests were estimated. After controlling for the effects of age and sex, Black children maintained statistically lower mean scores on the self-regulation scale when compared to Hispanic children (mean difference = -0.757, *F* = 13.72, Bonferroni corrected *p* = 0.0022) and when compared to Other children (mean difference = -0.801, *F* = 10.67, Bonferroni corrected *p* = 0.0093).

The second model presented in Table 2, labeled “Select Risk Factor Model,” reveals the impact of the racial category variables on self-regulation after 11 of the 12 risk factors were entered into the equation. Consistent with Hypothesis 1, the effect size for all but one of the racial category variables (*Asian*) decreased in magnitude between model 1 and model 2. Specifically, the mean difference in self-regulation between Black children and White children diminished to 1.006 in model 2 compared to the difference of 1.338 that was observed in model 1. This indicates that roughly 25 percent of the mean difference in self-regulation observed between White children and Black children was mediated by the pre-/perinatal risk factors. Indeed, the indirect effect of the *Black* coefficient was -0.323 and was statistically significant (standard error = 0.063, *p*<0.001, two-tailed). (Indirect effects were estimated using the structural equation modeling (“sem”) package and the “teffects” post-estimation command available in *Stata 14*.) A similar pattern of results was observed for the *Hispanic* coefficient. The White-Hispanic difference in self-regulation was no longer statistically significant in model 2 (*p*>0.05, two-tailed). The indirect effect of *Hispanic* on *Self-regulation* was -0.348 and the effect was statistically significant (standard error = 0.079, *p*<0.001, two-tailed).

As noted above, Black children had significantly lower mean scores on the self-regulation scale compared to Hispanic children and Other children. When these mean differences were analyzed in model 2, the difference between Black and Hispanic children remained (mean difference = -0.787, standard error = 0.208, *F* = 14.28, Bonferroni corrected *p* = 0.0017, indirect effect = -0.026, standard error = 0.053, *p* = 0.627) but the difference between Black and Other children was reduced to non-significance (mean difference = -0.683, standard error = 0.240, *F* = 8.13, Bonferroni *p* = 0.032, indirect effect = 0.118, standard error = 0.056, *p* = 0.036). Collectively, the findings from model 2 suggest that a portion of the racial differences in mean levels of self-regulation that was observed in Table 1 can be attributed to racial differences in exposure to the pre-/perinatal risk factors. These findings lend support for Hypothesis 1 but they do not fully account for the racial differences in self-regulation. Notably, the Black-Hispanic difference in self-regulation was not affected by the inclusion of the risk factors and the White-Asian difference grew larger.

Careful consideration of model 2 will reveal that the SES variable was not included. SES was not included in model 2 so that the effects of the other 11 pre-/perinatal risk factors could be observed. Each of the pre-/perinatal risk factors is likely to be intertwined with SES. Including SES in the regression model could, therefore, lead to problems with multicollinearity that would mask certain influences. Thus, we opted to include SES in model 3 after the other risk factors had already been entered in model 2. As can be seen from the results, many of the substantive conclusions remained unchanged from model 2 to model 3. For instance, the mean difference between White and Black children was reduced when compared to the coefficient presented in model 1. In model 3, the indirect effect of *Black* was -0.483 (standard error = 0.092, *p*<0.001), which was roughly a 37% reduction over the difference observed in model 1. This result suggests that a portion of the mean differences in self-regulation between White and Black children can be explained by the group differences in SES. Note also that the difference between White children and Asian children remained statistically significant in model 3 (*F* = 22.37, Bonferroni corrected *p*<0.0001), as did the difference between Black children and Hispanic children (mean difference = -0.707, standard error = 0.217, *F* = 10.61, Bonferroni corrected *p* = 0.0095).

Two other points about the results in model 3 are worth bearing in mind. First, the impact of *Maternal Age at Childbirth* remained statistically significant even after SES was included as a covariate. Second, the effect of *Maternal Education at Childbirth* was no longer statistically significant once SES was controlled. This latter result was expected given the conceptual overlap between SES and educational outcomes. Moreover—and as was noted above—the SES variable was calculated by combining educational and occupational measures into a single scale. (It is possible that certain risk factors will have a non-linear effect on self-regulation. We explored this possibility by entering quadratic versions of the following variables: *Prenatal Care*, *Gestation Week*, *and Birth Weight*. None reached statistical significance in the Full Risk Factor Model [*Prenatal Care*^*2*^ *b* = 0.00026, standard error = 0.0016, *p* = 0.872; *Gestation Week*^*2*^ *b* = 0.000035, standard error = 0.0035, *p* = 0.992; *Birth Weight*^*2*^ *b* = -0.00083, standard error = 0.0010, *p* = 0.422])

The fourth model presented in Table 2 is a trimmed version of the full risk factor model (i.e., model 3). Due to the co-occurrence of certain pre-/perinatal risk factors, we were concerned that the results presented in model 3 may be sensitive to multicollinearity between the risk factors. (We thank an anonymous reviewer for raising this point) In order to assuage these concerns, a stepwise regression model was estimated where risk factors with a *p*-value of 0.20 or lower in model 3 were retained and all others were omitted. The coefficient estimates for the retained risk factors are provided in the last column of Table 2 (i.e., model 4). The most important point to take away from this model is that the overall conclusions gleaned from the full risk factor model remain when the stepwise model was estimated. In short, *Maternal Age at Childbirth* and *SES* were statistically significant predictors of the child’s self-regulation. Moreover, the racial differences in self-regulation were substantively unchanged from model 3 to model 4. Finally, and somewhat uniquely, the effect of *Gestation Week* emerged as an independent predictor of self-regulation. The results suggest that each one-unit increase in gestation week was associated with a 0.057 point gain in self-regulation on average.

The final step to the analysis was to analyze whether the risk factors had a greater impact on mean self-regulation scores for White children and Black children compared to the other racial categories. Only White and Black children were analyzed in these models because they were the two racial groups that showed consistent mean differences in self-regulation in Table 1 and in Table 2. Put differently, it was unnecessary to analyze the interaction between *Asian* and the risk factors because these children did not present significantly different mean scores on self-regulation when compared to the other racial categories (except for when they were compared with White children).

In order to explore these relationships, a series of regression models were estimated where self-regulation was the dependent variable and age, sex, and the racial category of focus were the covariates (the racial category coefficient can now be interpreted as the difference between White or Black children and all other children). In addition, each of the risk factors, along with an interaction term between race and the risk factor (e.g., Risk Factor X White), were included in the model one at a time. There are two panels of information provided in Table 3. The top panel presents the coefficients from models where White children were compared against all other children. The bottom panel re-estimated the models, but this time Black children were compared against all other children. Due to the large number of coefficients provided in Table 3, we have bolded the Risk Factor X Race interactions that emerged as statistically significant. A quick glance at Table 3 reveals that only three of these interactions were statistically significant, and only one of those three was statistically significant at the 0.01 alpha level. Given the large number of models that were estimated (22), it was anticipated that one or two interactions may emerge as statistically significant purely by chance under the 0.05 alpha criterion. Considering that the statistically significant interactions do not follow a discernable pattern, we caution against any strict interpretations of these results because of the high likelihood that they are false-positive results. This is not to say that the three interactions that were statistically significant *are* false-positives, just that they are more likely to be so due to the large number of tests that were run. With this in mind, we are hesitant to conclude that there is any support for Hypothesis 2. (Given the known developmental differences for male and female children, it was important to explore whether the impact of the risk factors varied across sex. As a result we estimated a series of regression models where the child’s sex was included as a moderator of the impact of each risk factor. There was little indication that the effect of the risk factors varied across sex, but we are hesitant to put much weight on these findings because several of the statistical models showed signs of parameter indeterminancy due to a nonsymmetric variance matrix.)

**Table 3:** OLS Regression of Self-regulation on Risk Factors X Race Interactions (*n* ≈ 9,850).

	Model: Maternal Age	Model: Maternal Education	Model: Prenatal Care	Model: Gestation Week	Model: Birthweight/100	Model: Apgar	Model: Plural Births	Model: Vaginal Delivery	Model: Eclampsia	Model: Meconium	Model: Maternal Tobacco Use
	b/se	b/se	b/se	b/se	b/se	b/se	b/se	b/se	b/se	b/se	b/se
*White Children vs*. *All Other Children*											
White = 1	0.528(0.697)	-1.448(0.801)	0.664(0.472)	3.925*(1.675)	0.658(0.537)	0.464(1.596)	0.315(0.337)	0.806**(0.247)	0.817**(0.142)	0.834**(0.150)	0.950**(0.140)
Risk Factor	0.069**(0.016)	0.078*(0.030)	0.005(0.019)	0.099**(0.029)	0.033**(0.012)	0.113(0.124)	-0.457(0.242)	0.159(0.228)	-0.876(1.199)	0.241(0.360)	-0.308(0.309)
Risk Factor X White	0.005(0.024)	0.153**(0.056)	0.012(0.037)	-0.081(0.043)	0.003(0.016)	0.040(0.181)	0.489(0.280)	0.017(0.265)	0.203(1.680)	-0.305(0.558)	-0.678(0.391)
*Black Children vs*. *All Other Children*											
Black = 1	-0.579(0.634)	-2.352*(1.004)	-0.822(0.489)	-5.882**(2.058)	-1.867**(0.669)	0.189(1.512)	-0.395(0.412)	-0.684*(0.295)	-1.106**(0.170)	-1.105**(0.177)	-1.204**(0.165)
Risk Factor	0.076**(0.014)	0.154**(0.023)	0.021(0.019)	0.034(0.027)	0.031**(0.009)	0.157(0.114)	0.004(0.158)	0.236(0.147)	-0.214(1.023)	0.160(0.290)	-0.752**(0.253)
Risk Factor X Black	-0.014(0.023)	0.107(0.077)	-0.025(0.042)	0.125*(0.054)	0.026(0.021)	-0.146(0.169)	-0.698*(0.319)	-0.588(0.317)	-1.743(1.382)	-0.218(0.520)	0.604(0.619)

Standard errors in parentheses; All estimates corrected for the survey design features of the ECLS-B study; All models control for the child's age (wave 2) and the child's sex; White children are compared against all others in the first set of equations; Black children are compared against all others in the second set of equations.

*p <.05;

**p <.01 (two-tailed).

## Discussion

The findings from the current study suggest that pre- and perinatal risk factors are related to the development of self-regulation in early childhood. Differential exposure to these risk factors offers a partial explanation for the observed differences in self-regulation between White children and Black children, between White children and Hispanic children, and between Black children and Other children. Thus, we find support for Hypothesis 1, the exposure level hypothesis, but we caution that support was only partial. Indeed, only two of the mean differences that appeared in Table 1 were reduced to non-significance in Table 2 (the mean self-regulation differences between White children and Hispanic children and the mean self-regulation differences between Black children and Other children). Alternatively, the mean level difference in self-regulation between White and Black children was reduced but remained statistically significant after controlling for the pre-/perinatal risk factors. The difference between White and Asian children was not explained by exposure to the risk factors, nor was the difference between Black and Hispanic children. These latter results suggest there are additional risk factors that should be considered when analyzing the development of self-regulation among minority groups in the U.S.

After carefully considering the various combinations of Risk Factor X Race interactions, we concluded that there is little-to-no support in the present analysis for Hypothesis 2. Recall this hypothesis suggested certain racial groups are more susceptible or more sensitive to the pre-/perinatal risk factors. The present findings do not support this conclusion, at least not for the risk factors that were analyzed herein. This result may be promising because, when combined with the support gleaned for Hypothesis 1, our study suggests racial differences in levels of self-regulation can be (partially) addressed by minimizing *exposure* to pre-/perinatal risk factors. We find little reason to be concerned that a risk factor is more influential for one group over another, which simplifies the problem to issues surrounding exposure and not susceptibility. An analogy may help to clarify these points. The medical community has long recognized the need for (and complexities of) personalized medical approaches [56–57], where treatments are tailored to the individual based on his/her known susceptibilities and risks. While we see this as an important development, the present findings suggest broad policies that serve to reduce *all* children’s exposure to pre-/perinatal risk factors will likely represent a step in the right direction for closing the gaps identified here.

With the above points in mind, two implications stem from the present findings. First, it is important to note that each of the risk factors analyzed are potentially malleable. This means that differential exposure to the risk factors might be mitigated via the implementation of empirically robust public policies. One potential issue is minority mothers’ reduced—when compared to White mothers’—exposure to prenatal care in the first trimester [34]. This finding represents a starting point for public policy to take action: greater uniformity in access to prenatal health care is needed. Reducing the disparities in health care access may help to diminish the differential rates of exposure to the risk factors that were spotlighted above [46, 58]. A second implication is that an educational campaign may alleviate the disparity in prenatal health care visits observed across the racial categories (see Table 1). Such a campaign might focus on informing future parents of the benefits of prenatal health care—or the risks of ignoring health care. Women should be informed of how to gain access to prenatal care, where facilities are located, and how to determine what their insurance covers. Building on prior research [34, 46, 59], the results reported here might justify such a campaign. We are reluctant, though, to suggest that educational campaigns and sweeping policies will completely eliminate the racial differences observed in the current study. Recall, that our findings suggested that only a portion of the gap in self-regulation between races was accounted for by pre-/perinatal factors. This point reminds us that racial differences in access to health care and racial differences in levels of self-regulation are likely to be connected in complex ways. Although we have attempted to simplify our findings by noting the most promising implications that flow from the results, readers must keep in mind that human development follows a complex trajectory that can be altered by any number of factors. Thus, while our results may seem straightforward, much work is still needed to fully understand the origins of racial differences in self-regulation. Only at that point can well-informed policy decisions be made.

Limitations of this research must be addressed. First, the data analyzed in the current study were drawn from the year 2001. This opens the possibility that more recent data would reveal a different pattern of results. Available evidence, however, does not appear to support this claim: Martin et al. [60] reported that disparities in prenatal care remained in 2007. Approximately 71 percent of all mothers received prenatal care during the first trimester compared to 59.2 percent of non-Hispanic Black mothers who received the same care. The disparity in prenatal health care has, however, been narrowing over the past few decades [35], which is a promising trend. A second limitation was that we were unable to fully account for the racial differences in self-regulation with the risk factors available in the ECLS-B study. We encourage future work to expand the list of pre-/perinatal risk factors to determine whether there are other influences that should be given careful consideration by would-be parents, policy makers, and public health officials. Third, some of the measures were gleaned from maternal self-reported information and others were taken from the child’s birth certificate. None of the measures were assessed by objective raters (with the exception of items that were reported on the birth certificate by medical staff [e.g., Apgar]). To the extent that mothers purposefully omit certain information, our findings may be affected by social desirability biases. Note that some of the most sensitive information was retrieved from the child’s birth certificate, thereby alleviating these concerns for select variables. We highlight these concerns so that readers take appropriate cautions when interpreting the findings.

## Supporting Information

S1 Table Correlation Matrix. (XLSX)
